# Next-generation sequencing reveals microRNA markers of adrenocortical tumors malignancy

**DOI:** 10.18632/oncotarget.16788

**Published:** 2017-04-03

**Authors:** Łukasz Koperski, Marta Kotlarek, Michał Świerniak, Monika Kolanowska, Anna Kubiak, Barbara Górnicka, Krystian Jażdżewski, Anna Wójcicka

**Affiliations:** ^1^ Department of Pathology, Medical University of Warsaw, Warsaw, Poland; ^2^ Laboratory of Human Cancer Genetics, Center of New Technologies, CENT, University of Warsaw, Warsaw, Poland; ^3^ Genomic Medicine, Medical University of Warsaw, Warsaw, Poland

**Keywords:** microRNA, adrenocortical carcinoma, cancer diagnostics, next-generation sequencing, NGS

## Abstract

**Background:**

Adrenocortical carcinoma is a rare finding among common adrenocortical tumors, but it is highly aggressive and requires early detection and treatment. Still, the differential diagnosis between benign and malignant lesions is difficult even for experienced pathologists and there is a significant need for novel diagnostic methods. In this study we aimed to reveal a complete set of microRNAs expressed in the adrenal gland and to identify easily detectable, stable and objective biomarkers of adrenocortical malignancy.

**Methods:**

We employed next-generation sequencing to analyze microRNA profiles in a unique set of 51 samples, assigned to either a learning dataset including 7 adrenocortical carcinomas (ACCs), 8 adrenocortical adenomas (AAs) and 8 control samples (NAs), or a validation dataset including 8 ACCs, 10 AAs and 10 NAs. The results were validated in real-time Q-PCR.

**Results:**

We detected 411 miRNAs expressed in 1763 length isoforms in the examined samples. Fifteen miRNAs differentiate between malignant (ACC) and non-malignant (AA + NA) tissue in the test set of independent samples. Expression levels of 6 microRNAs, miR-503-5p, miR-483-3p, miR-450a-5p, miR-210, miR-483-5p, miR-421, predict sample status (malignancy/non-malignancy) with at least 95% accuracy in both datasets. The best single-gene malignancy marker, miR-483-3p, has been validated by real-time RT PCR.

**Conclusions:**

As a result of the study we propose clinically valid and easily detectable biomarkers of adrenocortical malignancy that may significantly facilitate morphological examination. Since microRNAs can be detected in blood, the study brings tools for development of non-invasive diagnostics of adrenocortical carcinomas.

## INTRODUCTION

Adrenal tumors occur with a population frequency estimated at 4% [1, 2], and due to the growing use of imaging techniques, numerous patients with incidental findings are referred for further diagnostics. However, malignant lesions, i.e. primary adrenocortical carcinomas (ACCs) occur very rarely, affecting 0.5-2 persons/million [3]. The histological diagnosis of adrenocortical tumors is difficult and the distinction between benign adrenocortical adenomas (AAs, AAs) and ACCs poses a serious challenge. Currently, the Weiss system is the most widely used for classification of adrenocortical tumors. The system is based on 9 microscopic features: structural (necrosis, diffuse architecture, and portion of clear cell component), cytological (nuclear atypia, atypical mitosis, and mitotic index), and related to tumor invasiveness (vascular, sinusoidal and capsular invasion) [4, 5]. The presence of 3 or more Weiss criteria favors diagnosis of ACC, but their classification is subjective and therefore difficult even for experienced pathologists. Although ACCs are rare, they are characterized by a high mortality, as a 5-year survival is estimated at 20-40% [6, 7]. It is thus vitally important to identify sensitive, specific and inter-observer bias-free molecular markers allowing for proper classification of adrenocortical tumors, what is in agreement with recent approaches [8]. Among the possible molecules, microRNAs are emerging as the most promising markers, mainly due to their high specificity and stability in various biological material [9].

MicroRNAs (miRNAs) are short, non-coding RNAs that inhibit the expression of protein coding genes through binding to complementary sequences in their transcripts [10]. It is estimated that microRNAs regulate the expression of at least half of the human protein-coding genes including oncogenes and tumor suppressors [11, 12, 13] and this phenomenon might be more prevalent due to the exsitence of numerous length isoforms of a single microRNA – isomiRs [14, 15, 16]. IsomiRs may originate from imperfect specificity of cleavage of microRNA precursors or from trimming or extension of mature miRs [17]. Deregulation of miRNAs is observed in many cancers [18, 19], leading to aberrant expression of target transcripts. MicroRNAs are widely investigated as possible diagnostic tools, and this clinical utility of miRNAs is possible due to their unique biological properties: tissue- and disease-specific expression profiles [20, 21] and high stability, which allows for detection and reliable measurement of miRNAs in various biological materials, including fine-needle aspiration biopsy (FNA) [22], archived formalin-fixed and paraffin-embedded samples [9] or serum [23].

MicroRNA-based diagnostic and prognostic tests have been proposed for many human cancers [24, 25, 26], to mention lung cancer [27, 28], hepatocellular [29] and thyroid carcinoma [30]. However, to date there is no equivocal information on microRNAs that distinguish between malignant and non-malignant adrenocortical tumors. Various studies brought information on different sets of microRNAs deregulated in ACCs compared with non-malignant samples, with overexpression of miR-483p and downregulation of miR-195 as the most commonly observed markers of malignancy, while the data on other miRNA is inconsistent [31, 32, 33, 34, 35, 36]. This discrepancy results most probably from the methods used in the studies, such as microarrays or real-time PCR quantification that do not allow for thorough analysis of all the miRNAs expressed in adrenal cortex and aberrant in adrenocortical tumors. To identify specific and comprehensive microRNA signatures of adrenocortical tumors, we employed next-generation sequencing, a method that allows for simultaneous analysis of sequences and expression levels of all microRNAs present in the analyzed tissue. This analysis led to identification of numerous length isoforms of the expressed microRNAs, and a set of microRNAs that distinguish between malignant and non-malignant adrenocortical lesions with at least 95% accuracy.

## RESULTS

### MicroRNA read numbers

Over 130 million reads were obtained for the analyzed samples after demultiplexing, indicating an average of 5.7 million (M) reads per sample with a mean read number of 9.5M, 5.9M and 2.2M in the ACC, AA and NA groups, respectively. An average of 452 thousand (k) reads were aligned to the sequences of mature microRNAs from miRBase with a mean number of 465k, 658k and 234k reads in the ACC, AA and NA groups, respectively. Differences in number of reads were significant between ACC and NA (p=0.0003 and p=0.04 regarding total and aligned number of reads respectively) as well as between AA and NA (p=0.001 and 0=0.03). The numbers of total and aligned reads for each sample are provided in Supplementary Table 1.

### MicroRNA expression in adrenal cortex and adrenocortical tumors

The analysis of the learning dataset revealed that 411 out of 2042 mature miRNAs annotated in miRBase v19 were significantly expressed in adrenal cortex tissue (RPM ≥5 in at least 50% of samples within any of the three studied groups, ACC, AA or NA, Supplementary Table 2). The levels of most highly expressed microRNAs exceeded a median of 50,000 RPM in all sample types. These miRNAs included miR-486-5p (median expression 159,078 RPM), miR-10b-5p (median expression 100,035 RPM), miR-22-3p (median expression 58,453 RPM), miR-181a-5p (median expression 55,216 RPM) and miR-26a-5p (median expression 50,562 RPM) (Supplementary Table 2). These microRNAs potentially play an important biological role in adrenal cortex.

### MicroRNAs are expressed in numerous length isoforms

**Figure 1:**
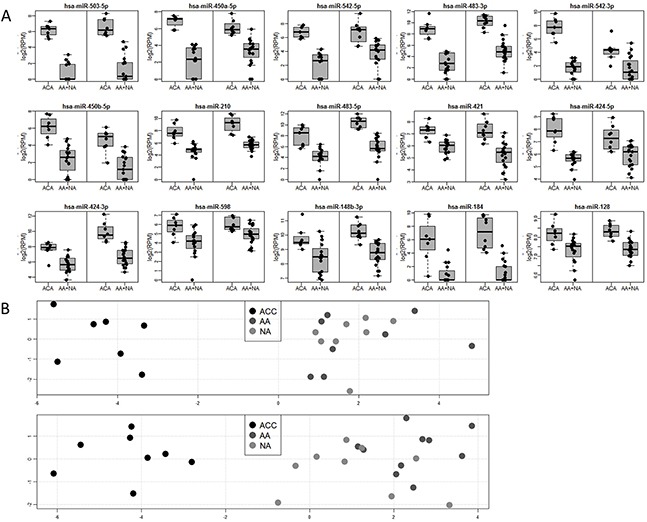
**(A)** Boxplots for 15 microRNAs significantly deregulated (FDR<0.05) in the comparison between malignant and non-malignant tissue in the learning (left box) and validation (right box) datasets. Abbreviations: ACC, adrenocortical carcinoma; AA, adrenocortical adenoma; NA, normal adrenal cortex. **(B)** Principal component analysis (PCA) showing global differences between samples based on expression of 15 deregulated microRNAs significantly deregulated (FDR<0.05) in the comparison between malignant and non-malignant tissue in learning (top) and validation (bottom) datasets. X-axis: PC1, Y-axis: PC2 significantly deregulated (FDR<0.05) in the comparison between malignant and non-malignant tissue

Individual miRNA genes produced several mature miRNA molecules that differed in length, called isomiRs. The analysis revealed that even though the adrenal tissue exhibited significant expression of only 411 microRNAs, they exist in 1763 various length isoforms (Supplementary Table 3). The identified microRNAs had up to 12 detected isoforms i.e. isoforms expressed in at least half of samples of the studied group at the level exceeding 1% of the total expression of a particular miRNA, and most microRNAs had 3-4 isoforms (Figure 1). The average number of isoforms per miRNA is 3.76±1.98, 4.24±1.91, 3.34±1.83 for ACC, AA, and NA, respectively. The distribution of expressed isomiRs per miRNA differs between tissue types: two or more isoforms produced by 88.2%, 96.1%, and 83.1% of miRNAs in ACC, AA, and NA, respectively (p-value 3.8 x10^−7^). The analysis revealed that the reference miRNA sequence was completely absent in 4.2 – 10.1% of the identified microRNAs (depending on the tissue type). Accordingly, for 38.4 – 42.7% of microRNAs it was not the most prevalent miRNA sequence (Supplementary Table 3).

### MicroRNA isoforms have unique seed regions

**Figure 2:**
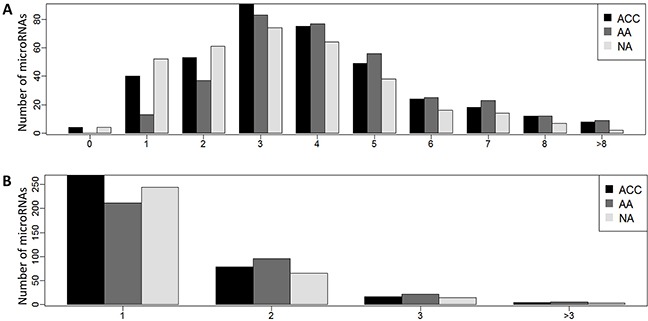
**(A)** Distribution of isomiRNAs and **(B)** seed sequences per miRNA in adrenocortical carcinoma (ACC), adrenocortical adenoma (AA), and normal adrenal cortex (NA).

Recognition of target genes depends on the microRNA seed sequence that can be changed due to alterations in microRNA length. Thus, the proper understanding of the role of microRNAs in regulation of the tissue transcriptome requires identification of all the expressed seed sequences, i.e. identification of isomiRs whose seed region is changed when compared to the miR's canonical counterpart. The analysis showed that 1763 adrenocortical isomiRs comprised 520 various seed sequences, of which 320 (61.5%) were canonical and 200 (38.5%) were novel seeds (Supplementary Table 4). Most microRNAs produced isoforms with the same seed sequence, however, 101 miRs (27.3%), expressed in ACC, 124 miRs (37%) expressed in AA, and 84 miRs (25.6%) expressed in NA produced isomiRs with two or more alternative seed regions (Figure 2) each targeting a unique set of target genes.

### Expression of microRNAs is deregulated in adrenocortical tumors

The Kruskal-Wallis test, performed on the learning dataset, revealed that 89 among 411 miRNAs were significantly expressed in adrenal cortex. These microRNAs were selected for validation in the independent set of samples (validation dataset). Expression of 21 among the selected miRNAs differed in the comparison of 3 studied groups at the significance level of FDR<0.05 in the test dataset (Table 1). Post-hoc analysis revealed that microRNAs top upregulated in carcinoma versus normal tissue included miR-509-5p, miR-184 miR-503-5p, miR-483-3p and miR-210 with at least 10-fold difference between both datasets. Top miRNAs upregulated in ACC compared with adenoma included miR-184, miR-483-3p, miR-542-3p, miR-509-5p, miR-503-5p, miR-483-5p, miR-450b-5p and miR-210 with at least 10-fold in both datasets. Interestingly, 13 among these miRNAs were positively validated (p<0.05 in both datasets) in ACCs vs NAs, 16 in AAs vs NAs and only 1 microRNA, miR-34a-5p, was deregulated between non-malignant AA and NA samples.

**Table 1:** Expression levels of 21 microRNAs significantly deregulated (FDR<0.05 in both learning and validation datasets) in the comparison of adrenocortical carcinoma (ACC), adrenocortical adenoma (AA) and normal adrenal cortex (NA)

	MEDIAN						Kruskal				p value of post-hoc pairwise test (Nemenyi - tests)						Pairwise fold change					
micro RNA name	Learning dataset			Test dataset			Learning dataset		Test dataset		Learning dataset			Test dataset			Learning dataset			Test dataset		
	ACC	AA	NA	ACC	AA	NA	p-value	FDR	p-value	FDR	ACC vs NA	ACC vs AA	AA vs NA	ACC vs NA	ACC vs AA	AA vs NA	ACC vs NA	ACC vs AA	AA vs NA	ACC vs NA	ACC vs AA	AA vs NA
**hsa- miR-184**	66.65	0.78	0.00	216.1	3.03	0.00	0.003	0.021	0.000	0.003	0.015	0.035	0.951	0.000	0.011	0.532	74.03	161.369	0.459	81.363	75.492	1.078
**hsa-miR-483-3p**	483.2	4.08	23.34	1334.3	22.33	56.22	0.001	0.014	0.000	0.003	0.022	0.001	0.565	0.027	0.000	0.349	47.44	114.759	0.413	9.947	59.073	0.168
**hsa-miR-542-3p**	212.75	3.28	3.81	20.14	0.00	4.63	0.001	0.014	0.001	0.008	0.008	0.003	0.934	0.305	0.001	0.103	73.15	94.502	0.774	3.007	17.711	0.170
**hsa-miR-509-5p**	46.52	0.00	0.00	13.22	0.00	0.00	0.000	0.014	0.003	0.018	0.007	0.031	0.874	0.015	0.008	0.993	N/A	64.746	N/A	28.563	232.056	0.123
**hsa-miR-503-5p**	81.66	0.00	0.00	72.76	2.59	0.00	0.000	0.014	0.000	0.003	0.006	0.011	0.978	0.001	0.002	0.984	66.23	41.792	1.585	29.051	27.649	1.051
**hsa-miR-483-5p**	374.1	10.92	27.56	1687.8	43.78	113.3	0.000	0.014	0.000	0.003	0.099	0.000	0.183	0.021	0.000	0.351	11.104	32.581	0.341	12.793	31.489	0.406
**hsa-miR-450b-5p**	75.10	3.69	8.95	33.64	1.12	3.10	0.001	0.014	0.001	0.009	0.046	0.001	0.376	0.018	0.002	0.835	8.49	21.414	0.397	6.561	11.363	0.577
**hsa-miR-542-5p**	110.9	6.01	7.41	135.76	18.36	18.19	0.001	0.014	0.004	0.020	0.005	0.005	1.000	0.011	0.015	0.983	18.43	15.791	1.167	8.717	8.109	1.075
**hsa-miR-450a-5p**	139.7	7.27	2.24	58.89	8.51	18.19	0.001	0.014	0.000	0.003	0.002	0.012	0.842	0.036	0.000	0.333	19.73	15.062	1.310	3.969	9.572	0.415
**hsa-miR-210**	192.7	29.94	28.43	629.9	52.59	49.38	0.001	0.014	0.000	0.003	0.006	0.005	0.999	0.004	0.001	0.941	10.06	10.989	0.915	12.469	14.536	0.858
**hsa-miR-424-5p**	231.7	58.12	48.92	159.0	35.31	80.48	0.001	0.014	0.004	0.020	0.001	0.021	0.587	0.428	0.005	0.133	6.73	5.753	1.170	2.213	3.902	0.567
**hsa-miR-98-5p**	2651.8	583.7	696.0	1007.6	389.5	584.0	0.001	0.014	0.004	0.020	0.008	0.003	0.934	0.515	0.005	0.104	4.55	5.181	0.877	1.709	3.214	0.532
**hsa-miR- 424-3p**	241.6	39.35	63.09	736.3	76.23	185.1	0.007	0.037	0.000	0.003	0.029	0.018	0.983	0.015	0.000	0.478	4.03	3.429	1.176	8.176	15.534	0.526
**hsa- miR-421**	159.2	52.80	68.99	136.1	39.98	57.48	0.002	0.020	0.001	0.007	0.027	0.005	0.822	0.024	0.001	0.662	2.52	2.740	0.921	2.967	4.848	0.612
**hsa-miR-34a-5p**	1040.6	780.3	81.15	336.0	744.2	269.8	0.002	0.016	0.008	0.036	0.003	0.667	0.033	0.919	0.063	0.017	10.53	2.239	4.704	1.251	0.582	2.150
**hsa-miR- 148b-3p**	711.0	547.7	313.2	1135.2	397.4	525.4	0.003	0.021	0.001	0.008	0.003	0.489	0.080	0.013	0.003	0.906	4.002	1.741	2.298	2.606	3.057	0.852
**hsa-miR-375**	36.3	107.1	11833.9	105.4	381.7	9027	0.009	0.044	0.000	0.005	0.019	0.898	0.052	0.000	0.175	0.088	0.003	0.686	0.004	0.007	0.033	0.203
**hsa-miR-129-2-3p**	0.00	0.00	24.76	0.00	3.06	31.95	0.001	0.014	0.008	0.036	0.026	0.998	0.024	0.009	0.335	0.221	0.007	0.434	0.016	0.026	0.267	0.099
**hsa-miR-30c-1-3p**	8.60	25.06	46.27	36.98	95.30	52.73	0.001	0.014	0.003	0.020	0.001	0.147	0.171	0.444	0.004	0.118	0.195	0.387	0.503	0.713	0.441	1.616
**hsa-miR-497-5p**	36.4	527.2	192.8	73.4	684.4	420.3	0.009	0.045	0.001	0.007	0.373	0.010	0.238	0.042	0.001	0.471	0.445	0.225	1.981	0.239	0.161	1.483
**hsa-miR-511**	0.00	5.97	4.26	0.00	8.03	10.86	0.004	0.024	0.008	0.036	0.116	0.006	0.513	0.021	0.026	0.989	0.109	0.066	1.661	0.136	0.153	0.885

Analysis of differences between samples from three analyzed groups was performed by Kruskal-Wallis rank sum test, followed by pairwise post-hoc analysis using Nemenyi test.

### Expression of microRNAs distinguishes between malignant and non-malignant tissue

**Figure 3:**
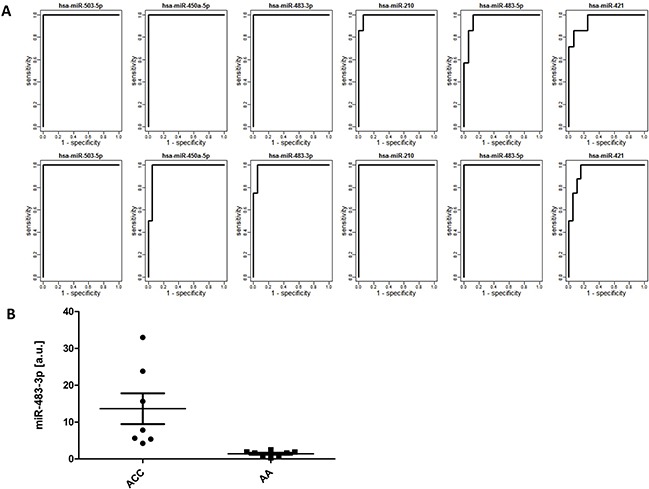
**(A)** ROC curves for 6 selected microRNAs in the learning (top) and validation (bottom) datasets. X-axis: 1-specificity, Y-axis:sensitivity; **(B)** Levels of miR-484-3p in 8 samples of adrenocortical carcinoma (ACC) and 8 samples of adrenocortical adenoma (AA) measured in triplicates in a real-time PCR Taqman assay, normalized against U6B. Data are expressed as median and the minimum-maximum range. The mean levels of miR-484-3p, calculated based on the 2^−ΔCt^ method were 13.41 in ACC vs 1.41 in AA samples, revealing a 9.7-fold increase in ACC (unpaired t-test, p=0.008).

Since the most important task in diagnosis of adrenocortical tumors is a sensitive and specific identification of malignancy, we tested whether microRNA profiles distinguish between malignant (ACC) and non-malignant (AA + NA) tissue. Based on the Welch t-test results performed on the learning dataset, 72 miRNAs were selected for validation (FDR < 0.05) and 15 of them were positively validated (FDR < 0.05) on the independent set of samples (Table 2, Figure 1). These highly specific microRNA profiles distinguish between malignant and benign adrenal cortex in both analyzed datasets, as illustrated by the PCA analysis (Figure 1). Most importantly, 6 miRNAs: miR-503-5p, miR-483-3p, miR-450a-5p, miR-210, miR-483-5p, miR-421 can serve as potent discriminators between malignant an non-malignant adrenal cortex tissue, as their expression in ACC is significantly higher than in non-malignant group, and area under ROC curve, which is a measurement of prediction accuracy, exceeds 95% in both analyzed datasets (Figure 3A). Although miR-503-5p was the only one with 100% AUC in both datasets we propose miR-483-3p, miR-483-5p and miR-210 to be considered the best candidates for molecular testing of adrenocortical carcinomas, as the mean expression of these microRNAs is high in both datasets (>325 RPM), and thus easily measurable, and the miRNAs are undetectable in non-malignant tissue. To additionally assess the usefulness of these microRNAs in a single-miR based Taqman probe diagnostics, we analyzed the expression of miR-483-3p in adrenocortical carcinoma and adenoma samples. The analysis confirmed the results obtained in NGS, revealing a mean 9.7-fold difference in the microRNA expression between the two sample sets (p=0.008) (Figure 3B), and proved the possibility of measuring the expression of miRNAs in a commonly used Taqman analysis.

**Table 2:** Expression levels of 15 microRNAs significantly deregulated (FDR<0.05 in both learning and validation datasets) between malignant (ACC) and non-malignant (AA + NA) tissue

microRNA name	MEAN				MEDIAN				T-TEST						AUC	
	Learning dataset		Test dataset		Learning dataset		Test dataset		Learning dataset			Test dataset			Learning dataset	Test dataset
	ACC	AA+ NA	ACC	AA+ NA	ACC	AA+ NA	ACC	AA+ NA	p value	FDR	Fold	p value	FDR	Fold	AUC	AUC
hsa-miR-503-5p	81.35	1.59	121.66	4.30	81.66	0.000	72.76	1.266	0.000	0.000	51.25	0.000	0.000	28.30	1.0	1.0
hsa-miR-483-3p	825.73	12.30	1441.25	81.47	483.22	6.724	1334.28	27.76	0.000	0.000	67.13	0.000	0.000	17.69	1.0	0.987
hsa-miR-450a-5p	124.42	7.28	83.04	14.48	139.75	5.111	58.89	11.77	0.000	0.000	17.08	0.000	0.000	5.74	1.0	0.974
hsa-miR-542-5p	124.74	7.33	183.58	21.89	110.86	6.224	135.76	18.19	0.000	0.000	17.01	0.000	0.003	8.39	1.0	0.914
hsa-miR-542-3p	318.75	3.87	32.82	6.14	212.75	3.657	20.14	2.081	0.000	0.000	82.46	0.001	0.004	5.34	1.0	0.868
hsa-miR-424-5p	315.22	50.81	195.17	68.10	231.70	51.29	158.97	74.03	0.001	0.008	6.20	0.004	0.018	2.87	1.0	0.829
hsa-miR-210	325.75	31.02	751.17	55.73	192.71	29.94	629.94	52.02	0.000	0.003	10.50	0.000	0.000	13.48	0.991	1.0
hsa-miR-450b-5p	97.74	8.03	31.74	3.76	75.10	6.012	33.64	2.243	0.000	0.001	12.16	0.000	0.000	8.44	0.982	0.941
hsa-miR-483-5p	425.19	25.67	1783.00	95.82	374.09	18.92	1687.80	51.18	0.000	0.006	16.56	0.000	0.000	18.61	0.964	1.0
hsa-miR-421	168.36	64.08	174.70	46.86	159.23	65.42	136.14	43.53	0.000	0.006	2.627	0.000	0.000	3.73	0.955	0.954
hsa-miR-184	264.21	2.60	323.38	4.14	66.65	0.000	216.12	0.000	0.005	0.035	101.5	0.000	0.001	78.16	0.938	0.980
hsa-miR-424-3p	241.53	65.18	1417.84	130.18	241.61	50.974	736.31	90.20	0.001	0.012	3.706	0.000	0.000	10.89	0.920	1.0
hsa-miR-128	319.03	171.32	333.34	174.25	297.04	184.283	315.31	165.42	0.008	0.046	1.862	0.000	0.000	1.91	0.866	0.961
hsa-miR-598	66.60	21.60	72.84	36.26	59.58	18.152	53.54	31.02	0.004	0.028	3.084	0.004	0.018	2.01	0.866	0.842
hsa-miR-148b-3p	1051.72	433.43	1324.71	468.92	711.03	357.624	1135.25	438.84	0.004	0.028	2.426	0.000	0.000	2.83	0.848	0.954

Testing was performed by Welch t-test. Area under curve indicates the diagnostic validity of each selected microRNA; AUC=1 indicates 100% specificity and 100% sensitivity which means that all samples were properly classified as malignant or non-malignant.

## DISCUSSION

This study, based on next-generation sequencing, identified 411 microRNAs expressed in the adrenal cortex, and revealed that these microRNAs exist in 1763 various length isoforms. The analysis was performed in a group of 51 samples, including adrenocortical carcinoma, adrenocortical adenoma and normal adrenal cortex. The samples were assigned to the learning and test groups, to assess diagnostic power of the proposed diagnostic microRNA panel. The study revealed that the levels of 15 microRNAs identify adrenocortical malignancy, and can be useful tools for identification of malignant lesions in adrenal cortex.

Adrenocortical tumors occur with a population frequency of 4%, but they are rarely malignant [3], and differential diagnosis between benign and malignant lesions relies on several clinical and morphological factors. Diagnostic imaging plays an essential role, as the sensitivity and specificity in predicting malignancy were 96% and 52%, respectively, for tumors ≥ 4cm, while for tumors ≥ 6cm the parameters reached 90% and 80% [40]. However, even in tumors smaller than 2cm, malignancy cannot be completely excluded [41]. Morphological diagnosis continues to play a key role in the final determination of the nature of the resected adrenal tumor, but in the absence of local invasion or distant metastases, differentiation between benign and malignant tumors can be problematic. The difficulties in identifying malignancy are reflected in the number of previously developed algorithms [42, 43]. The Weiss score (WS) is currently the most commonly used and the most validated system [4, 5] but its biggest drawback is the great subjectivity in the evaluation and relatively low reliability of certain microscopic criteria. Even though the least reliable parameters have been excluded [44], any attempt to seek new and better ways of differentiating benign and malignant adrenocortical tumors is fully justified.

Our study revealed that levels of 21 microRNAs differ in the comparison between the ACC, AA and NA samples, but the most interesting finding was identification of 15 microRNAs that could serve as markers of malignancy, as their levels differentiate between the malignant ACC and non-malignant AA+NA group. Among these, 6 miRNAs: miR-503-5p, miR-483-3p, miR-450a-5p, miR-210, miR-483-5p, miR-421 are the most potent indicators of malignancy as their expression is high and the prediction accuracy for each of them exceeds 95%. Moreover, miR-483-3p, miR-483-5p and miR-210 are highly expressed in carcinoma, and almost undetectable in non-malignant tissue. This was additionally confirmed in a QPCR assay analysing the expression of miR-483-3p. The family of miR-484, miR-210 and miR-503-5p were previously proposed as a good malignancy biomarkers in adrenocortical tumors [32, 36], what was confirmed in this study. However, our study showed that other previously proposed markers, including miR-139-3p and miR-675, have very low expression levels (∼10 RPM), thus their diagnostic utility is questionable. Similarly, our study showed that downregulation of previously reported miR-195 and miR-497 [32, 33, 35] was not statistically significant, moreover, compared with normal adrenal cortex, both miRNAs were indeed lowered in ACC, but upregulated in AA, which disqualifies them as potential biomarkers of malignancy. Our study showed that miR-34a-5p, previously proposed as a promising serum biomarker [45] is the only microRNA differentiating between the AA and NA samples.

Interestingly, ACC samples showed a general increase in total miRNA levels compared to AA and NA samples, which is contradictory to some observations in other cancers, where miRNA expression is lowered compared to normal tissue [46]. This phenomenon can be potentially explained by the fact that ACCs harbor numerous chromosomal amplifications [47, 48] leading to increased levels of genes encoded within the regions.

The study also led to identification of previously unreported microRNA isoforms expressed in the adrenal gland. We obtained expression profiles of canonical microRNAs and their newly identified isoforms whose aberrances potentially underlie initiation and progression of adrenocortical carcinogenesis. The recognition of mRNA by a microRNA depends on the “seed region” of a miR, comprising nucleotides 2-8 of mature molecule [49]. Sequence variations of many of the isomiRs are based on addition or deletion of nucleotides at their 5′end when compared to the reference miRNA, resulting in a change of the “seed region” and leading to recognition and regulation of distinct sets of target genes. Our study showed that over 38% of the seed sequences among the newly identified isomiRs differ from the canonical seed sequences deposited in miRBase, and, consequently regulate the expression of different target genes. This fact is of great importance for further studies on the role of microRNAs in the physiology and pathology of adrenal cortex.

As a result of the study we show a complete landscape of microRNA isoforms expressed in adrenal cortex and propose a clinically valid, objective and easily detectable microRNA markers of adrenocortical malignancy that may significantly facilitate morphological examination. Unfortunately, in this study, blood samples of ACC patients were not accessible, but the study brings tools for development of non-invasive diagnostics of adrenocortical carcinomas.

## MATERIALS AND METHODS

### Patient cohort and study design

Fifty-one archived formalin-fixed, paraffin-embedded (FFPE) tissue specimens were retrieved from the archives of Department of Pathology at the Medical University of Warsaw, Poland. The list includes 15 adrenocortical carcinomas (ACCs) having a Weiss score (WS) at least 5, and 18 conventional adrenocortical adenomas (AAs) with a Weiss score <1, and 18 normal, control adrenal cortex samples (NAs). The patients had undergone surgical resection of the adrenals between 2009 and 2015 and histopathological evaluation of the specimens was performed by two independent pathologists. The patients were randomly divided into the learning and test datasets. Clinicopathological information on the patients included in the learning dataset is provided in Supplementary Table 1. The material was retrieved according to the European procedures and regulations.

### MicroRNA extraction and expression analysis

Total RNAs were extracted from the FFPE specimens using InviTrap Spin Universal RNA Mini Kit (Stratec) and the quality and quantity of the obtained nucleic acid samples was assessed on NanoDrop2000 (Thermo Scientific, Wilmington, Delaware USA) and Bioanalyzer (Agilent, RNA 6000 Nano Kit, cat no: 5067-1511). 1μg of total RNA was used for next-generation sequencing experiment. cDNA libraries were prepared using TruSeq Small RNA Library Preparation Kits (Illumina). The obtained small RNA libraries were quantified on Bioanalyzer (Agilent, High Sensitivity DNA Kit, cat no: 5067-4627), pooled, and the appropriate range of cDNA fragments (120-150 bp) was extracted on a 3% gel using the BluePippin HT (Sage Science). The final length range of the library was verified on Bioanalyzer 2100 (Agilent) with the high sensitivity DNA kit and contained only the fraction of small RNAs. Small RNA sequencing was performed on a NextSeq 500 Instrument (Illumina) with the Next Seq500 High Output Kit, 75 cycles (Illumina), on 1.5pM library of cDNA.

The expression of miR-483-3p and a reference *U6B* gene was additionally analyzed in a real-time Q-PCR analysis with a specific Taqman probe (ID: 0023339; Life Technologies) on a Roche 480 LightCycler. The reaction was performed on 150ng of RNA according to the manufacturer's protocol and the expression of microRNA was calculated using the standard 2-ΔCt method

### Bioinformatic and statistical analysis

Raw data were demultiplexed and converted to FASTQ files using bcl2fastq v2.16.0.10 Conversion Software. Adapters were removed using cutadapt v1.7.1 software [37]. Obtained sequences with the length of 18-28 nucleotides were subject to further analysis as potential miRNAs. The sequences were mapped on the 2042 mature miRNAs sequences deposited in miRBase v19 [38] using Bowtie v0.12 [39] with the requirement of perfect matching. The numbers of mapped reads were counted for each miRNA, and RPM (Reads Per Million) normalization was performed for each analyzed sample. Differences between the total and aligned reads number between patients groups were analyzed by Wilcoxon test.

Analysis of differences between samples from the three analyzed groups was performed by Kruskal-Wallis rank sum test, followed by Nemenyi pairwise post-hoc test for significantly deregulated miRNAs. MiRNAs deregulated between malignant and non-malignant samples were identified by Welch t-test. The false discovery rate (FDR) was used to assess the multiple testing errors. To determine the predictive power of statistically significant miRNAs (FDR<0.05), the receiver-operating-characteristic (ROC) curves were constructed, followed by calculation of the area under curve. All statistical analyses were performed using R/Bioconductor environment. Principal component analysis (PCA) was used to visualize significant differences in the expression of 15 microRNAs between malignant (ACC) and non-malignant (AA + NA) tissue.

To detect all human isomiRs, an additional library of reference sequences was prepared by identifying the sequences of mature miRNAs, together with 5 flanking nucleotides, within the hairpins deposited in miRBase. The ratios of the number of isomiRs per miRNA among different sample types were computed using Pearson's chi-squared test. The expression of miR-483-3p in ACC and AA tissue samples was compared using an unpaired t-test.

## SUPPLEMENTARY TABLES











## Abbreviations

AA: adrenocortical adenoma
ACC: adrenocortical carcinoma
NA: normal adrenal cortex
NGS: next-generation sequencing
