# Supplementary material for: Next-generation sequencing reveals microRNA markers of adrenocortical tumors malignancy
Source: Oncotarget. 2017 Apr 3;8(30):49191–200. doi: 10.18632/oncotarget.16788 (PMC5564760; doi:10.18632/oncotarget.16788)
Supplement: Supplementary file 1 [file oncotarget-08-49191-s001.pdf]

Next-generation sequencing reveals microRNA markers of adrenocortical tumors malignancy

SUPPLEMENTARY TABLES

Supplementary Table 1: Characteristics of samples used in the study including patient age, diagnosis, stage of the disease and obtained numbers of microRNA reads

See Supplementary File 1

Supplementary Table 2: List of all microRNAs detected as expressed (RPM ≥5 in at least 50% of samples within any of the three studied groups) in the adrenal cortex. ACC – adrenocortical carcinoma, AA- adrenocortical adenoma, NA – normal adrenal cortex

See Supplementary File 2

Supplementary Table 3: List of all detected isoforms expressed in at least half of samples of the studied group at the level exceeding 1% of the total expression of a particular miRNA. ACC – adrenocortical carcinoma, AA- adrenocortical adenoma, NA – normal adrenal cortex

See Supplementary File 3

Supplementary Table 4: List of seed sequences from all expressed isoforms. ACC – adrenocortical carcinoma, AA- adrenocortical adenoma, NA – normal adrenal cortex

See Supplementary File 4
